# S08-2 Reproducibility and validity of four physical activity questionnaires in 18 European union member states: the European Union Physical Activity and Sport Monitoring System (EUPASMOS) project

**DOI:** 10.1093/eurpub/ckac093.041

**Published:** 2022-08-29

**Authors:** Maarten Schipper

**Affiliations:** National Institute for Public Health and the Environment, Utrecht, The Netherlands

**Keywords:** sedentary behavior, reproducibility, validity, questionnaire, accelerometer

## Abstract

**Background:**

The successful promotion of health-enhancing physical activity (HEPA) requires reliable and valid prevalence data of physical activity (PA) to enable the design, implementation and evaluation of effective and cost-effective policies. As part of the development of a new integrated monitoring framework in Europe, the reliability and validity of the Eurobarometer, European Health Interview Survey (EHIS), International Physical Activity Questionnaire (IPAQ) and Global Physical Activity Questionnaire (GPAQ) were studied in the ongoing European Union Physical Activity and Sport Monitoring System project (EUPASMOS) among 18 European Union member states.

**Methods:**

Physical activity (PA) and sedentary behavior (SB) were assessed among 80-100 adults per member state using the four questionnaires and the UKK RM42 accelerometer. Participants completed the questionnaires in random order and wore the accelerometer seven consecutive days on their right hip (during the day) and on their non-dominant wrist (overnight). Time spent in moderate-to-vigorous intensity physical activity (MVPA), sedentary behavior and adherence to the WHO PA guidelines were calculated based on the 5 methods. The reproducibility and validity of the four questionnaires were tested by using the Spearman's correlation coefficient (ρ) or Cohen's kappa coefficient (κ), dependent of the outcome measure.

**Results:**

Preliminary results from the Netherlands (N = 91) showed that the correlations for reproducibility of all four questionnaires varied between 0.51 and 0.63 for MVPA, between 0.30 and 0.60 for the WHO PA guidelines and between 0.65 and 0.82 for SB. All correlations were statically significant (p > 0.05). Significant correlations for validity of the questionnaires were found for MVPA (ρ = 0.22-0.43) and the WHO PA guidelines (ρ = 0.19-0.25). The only exception was the association between the EHIS questionnaire and the WHO PA guidelines. For SB, only GPAQ and the EHIS questionnaire showed significant associations (ρ = 0.31-0.32).

**Conclusions:**

First analyses of the Dutch data within the EUPASMOS project showed that the four commonly used questionnaires in Europe are fairly to strongly reliable methods depending on the outcome measure. The four questionnaires are fairly valid methods, except the Eurobarometer and IPAQ when examining SB. It is of interest to examine the quality of these questionnaires in all 18 member states for national and European policy makers.

